# Characterisation and expression profile of the bovine cathelicidin gene repertoire in mammary tissue

**DOI:** 10.1186/1471-2164-15-128

**Published:** 2014-02-13

**Authors:** Cormac J Whelehan, Anne Barry-Reidy, Kieran G Meade, P David Eckersall, Aspinas Chapwanya, Fernando Narciandi, Andrew T Lloyd, Cliona O’Farrelly

**Affiliations:** 1 Comparative Immunology Group, Trinity Biomedical Sciences Institute, Trinity College Dublin, Pearse Street, Dublin 2, Ireland; 2 Animal & Bioscience Research Department, Teagasc, Grange, Co Meath, Ireland; 3 Division of Animal Production and Public Health, Faculty of Veterinary Medicine, University of Glasgow, Bearsden Rd, Glasgow G611QH, UK; 4 Department of Production Animal Studies, Faculty of Veterinary Science, University of Pretoria, Onderstepoort, Pretoria, South Africa; 5 Current address: Department of Science and Health, Carlow Institute of Technology, Kilkenny Road, Carlow, Ireland

**Keywords:** Cathelicidin, Hidden Markov Model (HMM), Gene cluster, Locus, Tissue expression

## Abstract

**Background:**

Cathelicidins comprise a major group of host-defence peptides. Conserved across a wide range of species, they have several functions related to host defence. Only one cathelicidin has been found in humans but several cathelicidin genes occur in the bovine genome. We propose that these molecules may have a protective role against mastitis. The aim of this study was to characterise the cathelicidin gene-cluster in the bovine genome and to identify sites of expression in the bovine mammary gland.

**Results:**

Bioinformatic analysis of the bovine genome (BosTau7) revealed seven protein-coding cathelicidin genes, *CATHL*1-7, including two identical copies of *CATHL4,* as well as three additional putative cathelicidin genes, all clustered on the long arm of chromosome 22. Six of the seven protein-coding genes were expressed in leukocytes extracted from milk of high somatic cell count (SCC) cows. *CATHL5* was expressed across several sites in the mammary gland, but did not increase in response to *Staphylococcus aureus* infection.

**Conclusions:**

Here, we characterise the bovine cathelicidin gene cluster and reconcile inconsistencies in the datasets of previous studies. Constitutive cathelicidin expression in the mammary gland suggests a possible role for these host defence peptides its protection.

## Background

Rapid and effective responses to pathogenic challenge are essential for the survival of all living organisms. Production of host-defence peptides (HDPs), important effector molecules of the innate immune response, is key to effective anti-microbial activity at many sites, in many eukaryotic species. Cathelicidins are a family of host-defence peptides found in a diverse range of species, including hagfish [1], amphibians [2], fish [3], birds [4], snakes [5] and mammals ([6] and reviewed in [7]). Initially named antimicrobial peptides (AMPs) and categorised by their ability to act as endogenous antibiotics by disrupting microbial cell membranes [8], it is now becoming clear that their biological activity is multifunctional and includes chemotactic and immunoregulatory activities [9,10]. For example, the human cathelicidin peptide LL-37 (37 amino acids in length, beginning with two leucines), is chemotactic for neutrophils, monocytes and T cells [11] and has been shown to influence dendritic cell and monocyte function as well as TLR signalling [9].

**Figure 1:**
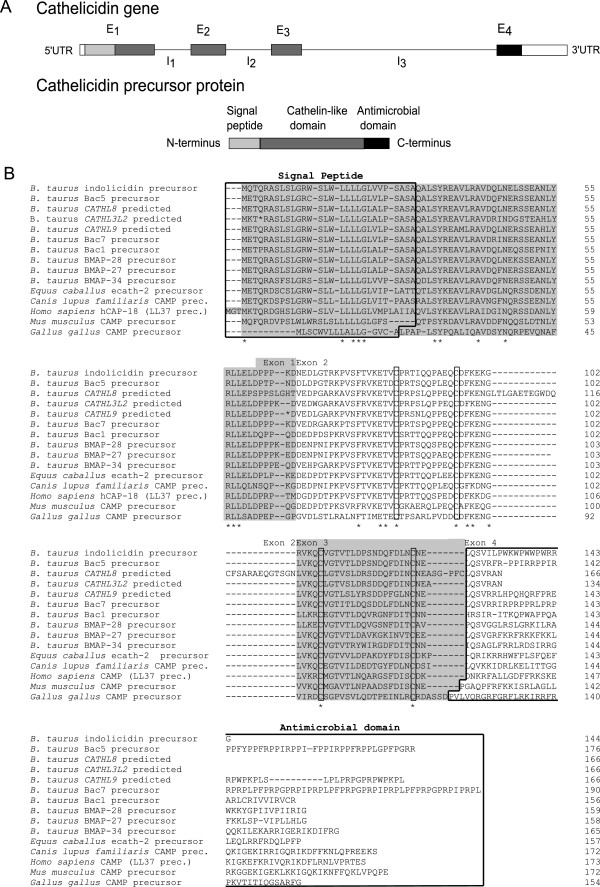
**Cathelicidin structure. A)** Cathelicidin genes are approximately 2 kilobases in size with a conserved four exon – three intron arrangement. Exons one, two and three code for the highly conserved N-terminal-coding region of the precursor protein known as the cathelin domain. Exon four codes for a highly variable domain with antimicrobial properties in the mature peptide. **B)** Alignment of bovine cathelicidin precursor proteins with those from human (*Homo sapiens*, hCAP-18), mouse (*Mus musculus*, CAMP), horse (*Equus caballus*, ecath-2), dog (*Canis lupus familiaris*, CAMP) and chicken (*Gallus gallus*, cathl2). (See Additional file 1 for details of the mature peptide encoded by each cathelicidin gene.) Conserved residues are indicated by the asterisk. Exons 1-3 are highly conserved between species with considerable variation in sequence within the exon 4 sequence within and between species. These peptides contain two disulfide bonds between cysteine residues C85-C96 and C107-C124.

Cathelicidins are so called because of a highly conserved N-terminal-coding region of the precursor protein known as the cathelin domain, followed by a highly variable domain which codes for peptides with antimicrobial activity (Figure 1A) [12]. The cathelin domain contains two disulfide bonds between cysteine residues C85-C96 and C107-C124 (Figure 1) and was given its name based on sequence similarity to a protein called cathelin, a member of the cystatin superfamily of cysteine proteinase inhibitors (cathe-l-in is an acronym for cathepsin L inhibitor) [13].

Although ubiquitous in mammals, the number of cathelicidin genes in the genome of any single species varies. For instance, while humans and mice have a single cathelicidin gene which codes for an α-helical mature peptide, multiple cathelicidin genes have been found in cattle and sheep [7,14-16]. In these latter species, the expanded cathelicidin repertoire also includes linear peptides whose mechanism of action may be different to the ancestral α-helical structures [7].

The bovine lineage was one of the first in which cathelicidins were discovered during studies of the antimicrobial activity of bovine neutrophil lysates [17]. In particular, indolicidin, which was first discovered in bovine neutrophils [18] has been shown to exert antimicrobial activity against a number of well-known pathogens, including *Staphylococcus aureus*[19]. Additional cathelicidin genes have been discovered in the bovine genome using molecular cloning strategies [20-23]. To date, at least seven distinct protein-coding cathelicidin genes have been identified in the bovine genome and are found in a single cluster on chromosome 22 [15]. Southern blot analysis of bovine genomic libraries identified a second copy of *CATHL4* and of *CATHL1*, as well as two related genes that were found to contain premature stop codons [23].

When tested for *in vitro* antimicrobial activity, cathelicidins rapidly killed a wide range of microorganisms (reviewed in [7,16]). BMAP-27 and BMAP-28 peptides, encoded by *CATHL6* and *CATHL5* respectively, display significant levels of antimicrobial activity against *E. coli* when assayed in milk from cows with mastitis [24] (see Additional file 1 for names of mature antimicrobial peptides encoded by the *CATHL* genes). The importance of cathelicidins *in vivo* can be inferred from their localisation at sites which are exposed to microbial invasion as well as in leukocytes. LL-37 has been detected in human neutrophils, mast cells, monocytes and macrophages [25-28]. Epithelial cells in skin, gut, lung, epididymis and mammary gland are other important sources of LL-37 [29-33], which is therefore thought to have a key role in host defence at sites that are in contact with the external environment [34]. *CATHL5* transcript has been detected in the mammary gland of healthy cows, but a significant increase in transcript levels in animals with a naturally-occurring intramammary infection was not observed [24].

The sequencing of the bovine genome has provided an opportunity for detailed study of the cathelicidins in a ruminant species. Initial examination of the complete cathelicidin cluster by Elsik *et al*. [35] indicated there were three additional cathelicidin genes, which they called cathelicidin 8, cathelicidin 9 and cathelicidin 10. Later Dawson *et al.*[36] searched for immune gene family expansions in artiodactyls and also identified three potential novel bovine cathelicidin genes, which they named *CATHL2L*, *CATHL3L1* and *CATHL3L2*. However although both groups identified 10 cathelicidins in the bovine genome, the two sets of results were not completely congruent, with differences in the positioning of the novel sequences relative to the known cathelicidins in the cluster. Here, we attempt to reconcile these differences by conducting a comprehensive annotation of the cathelicidin locus in *Bos taurus* using homology-based search methods of the BLAST family of programs [37] and the more sensitive Hidden Markov Models (HMM) approach [38]. We examined cathelicidin expression in somatic cells isolated from milk samples from animals with high somatic cell counts (SCC), a metric commonly used to detect intramammary infection (mastitis). We also used an *in vivo* model of mastitis to examine cathelicidin expression across several regions of the mammary gland.

## Results

### Characterisation of the bovine cathelicidin gene cluster

**Figure 2:**
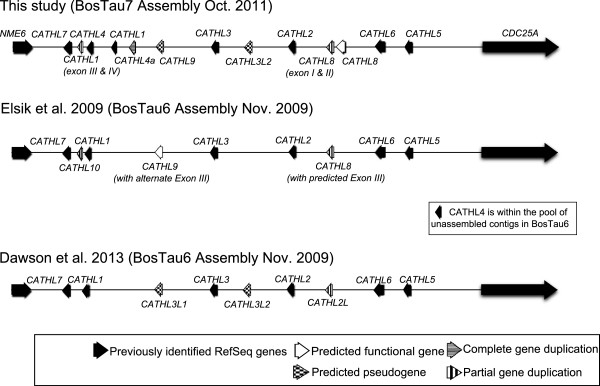
**Genomic organisation of the bovine cathelicidin gene cluster on bovine chromosome 22 (BTA22).** Comparison of recent annotations of the bovine cathelicidin cluster [33,34]. Our results are based on the BosTau7 (Baylor) genomic assembly, while the Elsik *et al.* and Dawson *et al.* results are based on the alternative BosTau6 (University of Maryland) assembly. Both assemblies are based on sequence data from the same Hereford animal. Genes predicted to be pseudogenised contain premature stop codons.

The BosTau7 assembly of the bovine genome provided us with the opportunity to reconstruct the full cathelicidin region for this species. A HMM profile was constructed based on the alignment of protein sequences corresponding to the seven known bovine cathelicidins. This was then used to search the sequenced bovine genome which had been translated in all six reading frames. Evidence for the presence of all seven protein-coding cathelicidin genes was found on chromosome 22 (BTA22) of the bovine genome BosTau7 assembly (Figure 2). Two copies of *CATHL4* were detected within this locus, as were second copies of exons 3 and 4 of *CATHL1*. This is in agreement with the results obtained by Scocchi *et al.*[21] using Southern blotting and indicates that our findings are not the result of genome assembly errors. All cathelicidin genes are clustered in an approximately 100 kb length of DNA, located on the long arm of chromosome 22 (22q24). The genes are localized on one strand and all are transcribed in the same direction.

This search led to the identification of 4 cathelin-containing motifs additional to the seven protein expressing genes already annotated: one intact gene, two genes containing premature stop codons and one partial gene duplication. Chromosomal location and strand orientation of the identified cathelicidins was determined using the BLAST-like Alignment Tool (BLAT) at the University of California, Santa Cruz genome browser (Figure 2).

**Figure 3:**
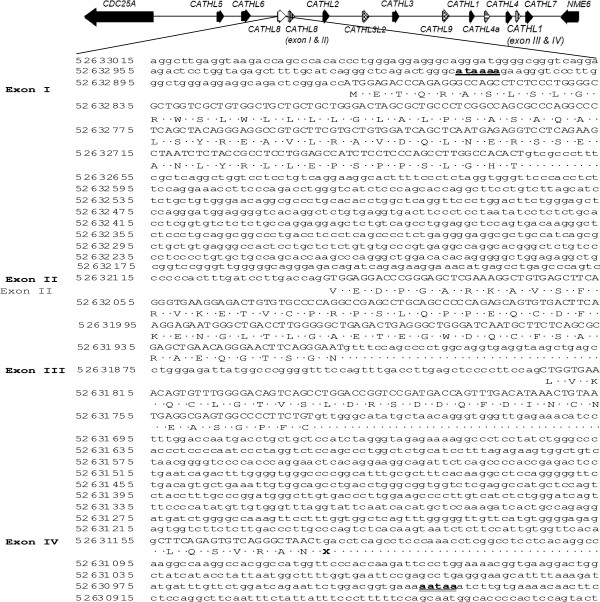
**Predicted structure of *CATHL8*.** Genomic DNA corresponding to the putative cathelicidin, *CATHL8*, was retrieved using BLAST-like Alignment Tool (BLAT) at the University of California, Santa Cruz genome browser (http://genome.ucsc.edu) and used for prediction of intron/ exon boundaries using GenScan (http://genes.mit.edu/GENSCAN.html). The coding sequence is in uppercase letters, noncoding sequences are in lowercase letters. The deduced amino acid sequence of the open reading frame is indicated in single letter code and the stop codon is indicated by an x. The TATA-box signal is in bold and underlined and the polyadenylation signal is in bold and double underlined. Line numbers represent genomic co-ordinates on the BosTau7 assembly.

Genomic DNA corresponding to these putative cathelicidins was used for prediction of intron/exon boundaries with GenScan software (Figure 3) before the additional predicted cathelicidin sequences were compared with those proposed by previous authors [35,36] (Figure 2). ClustalW, in MEGA 5.2 [39], was used to align the additional cathelicidin sequences found in our analysis with the *CATHL8*, *CATHL9* and *CATHL10* genes described by Elsik *et al.*[35], as downloaded from the Bovine Genome Database (http://www.bovinegenome.org[40]). *CATHL8* corresponds with the partial copy of the single intact gene we found. However the third and fourth exon of our predicted intact gene share a high level of sequence similarity with other bovine cathelicidins (Figure 1B), yet differ from that predicted by Elsik *et al*. Another copy of the first two exons of this putative gene lies adjacent. *CATHL9* is identical to one of the pseudogenes, with the exception of a tryptophan residue in place of the stop codon we have predicted, while our analysis also predicts a different fourth exon. *CATHL10* appears to be identical to the duplication of *CATHL4* we have annotated.

The same procedure was used to align the additional cathelicidin sequences found in our analysis with the Ensembl sequence IDs referenced by Dawson *et al.*[36] for *CATHL2L*, *CATHL3L1* and *CATHL3L2*. Based on these alignments, *CATHL2L* is 100% identical to the partial duplication of *CATHL8*, while *CATHL3L1* is 100% identical to the pseudogenised version of *CATHL9* we have predicted. The remaining pseudogene we found shares full sequence similarity with *CATHL3L2*. Based on these results, we named the predicted additional cathelicidin genes *CATHL8*, *CATHL3L2* and *CATHL9,* following the rule of priority (Figure 2). See Additional files 2 and 3 for the predicted structures of CATHL9 and CATHL3L2 respectively.

**Figure 4:**
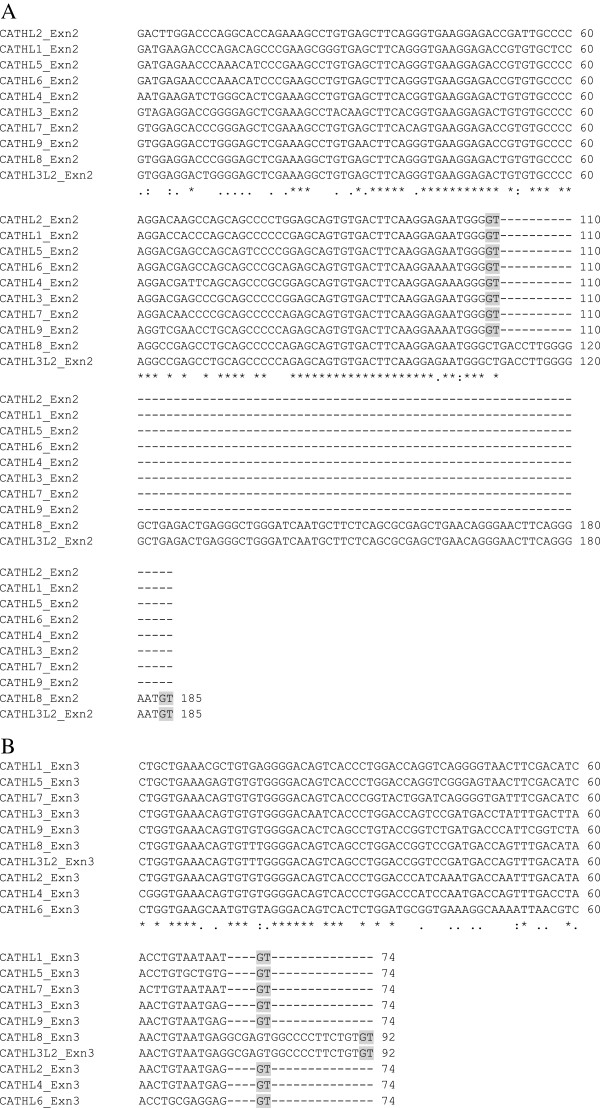
**Alternative 5′ splice junctions predicted for exon 2 and exon 3 of *CATHL8 *and *CATHL3L2*.** Genomic DNA corresponding to exon 2 and exon 3 of *CATHL*1-7, *CATHL8*, *CATHL3L2* and *CATHL9* was retrieved from the University of California, Santa Cruz genome browser (http://genome.ucsc.edu). These sequences were aligned using ClustalW, in MEGA5.2 [39]. **A)** Exon 2 alignment with donor sites (splice junctions) highlighted in grey. **B)** Exon 3 alignment with donor sites highlighted in grey. Conserved nucleotides are indicated by an asterisk.

*CATHL8* displayed all the characteristics of a functional cathelicidin including 2 kilobase size and a conserved four exon – three intron arrangement (Figure 3) with a TATA-box just upstream from the transcription start site and a polyadenylation signal located 54 bp from the stop codon (Figure 3). In addition, several potential recognition sites for transcription factors involved in the transcription of immune-related genes were found in the 5′ flanking region of this predicted gene (Additional file 4). However, alternative 5′ splice junctions were predicted, changing the 3′ boundary of exon 2 and exon 3 when compared to cathelicidins 1-7 (Figure 4A and B). Therefore, the predicted sequences and their intron-exon boundaries are not consistent with cathelicidins 1-7, and as a result, *CATHL8* does not display the canonical cysteine spacing that is invariably conserved among other cathelicidin family members [7].

**Figure 5:**
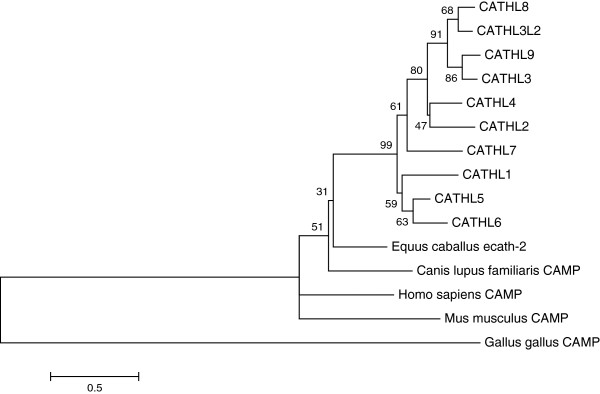
**Phylogram indicating evolutionary relationship between the bovine cathelicidin genes.** Maximum likelihood phylogram based on an alignment of the nucleotide sequences of the exons from the 10 bovine cathelicidin genes which have all four exons. Sequences were obtained from the BosTau7 genome assembly using the UCSC genome browser. A Tamura 3-parameter model with a gamma distribution of substitution rates among sites was selected as being the most appropriate using jModelTest2. Numbers at the nodes are the results of 10,000 bootstrap replicates. Scale bar is number of substitutions per site.

Phylogenetic analysis of all protein-coding and predicted bovine cathelicidins revealed that all the predicted putative cathelicidin genes had highest nucleotide identity to *CATHL3* (Figure 5). Bootstrap support is relatively low at all nodes because the cathelin-like domain, which is encoded by three out of the four exons, is so highly conserved, meaning the evolutionary signal is mostly coming from the relatively short but variable fourth exon.

### Expression of cathelicidin genes 1-8

Cathelicidins 1-7 were constitutively expressed in a range of tissues from multiple body systems (Table 1). However, we were unable to detect expression of the putative genes within cDNA of these tissues.

**Table 1:** qRT-PCR expression of cathelicidin genes 1-7 in a range of healthy bovine tissues

**Tissue**	***CATHL1***	***CATHL2***	***CATHL3***	***CATHL4***	***CATHL5***	***CATHL6***	***CATHL7***
Ovary	-	+	-	+	++	-	-
Fallopian Tube	-	-	-	-	++	-	-
Testis	++	+	-	+	+++	-	++++
Uterus	-	-	-	-	++	-	-
Rumen	+	-	+++	-	++	-	-
Small Intestine	-	+	+	-	-	-	-
Large Intestine	+	-	++	-	++	-	-
Liver	++	+	++	++	+++	-	++
Spleen	++	++	++	+	++	++	++
Lymph Node	++	+	-	-	++	++	-
PBMCs	-	-	+	-	-	+	-
Lung	+	+	-	+	+++	+	+
Mammary Gland	-	+	-	++	++	-	-

- denotes a lack of detectable gene expression, + denotes 2-20% mRNA expression relative to *GAPDH,* ++ equals 20-40%, +++ equals 40-60% and ++++ equals 60-80%.

### Cathelicidin gene expression in tissues from healthy mammary gland

**Figure 6:**
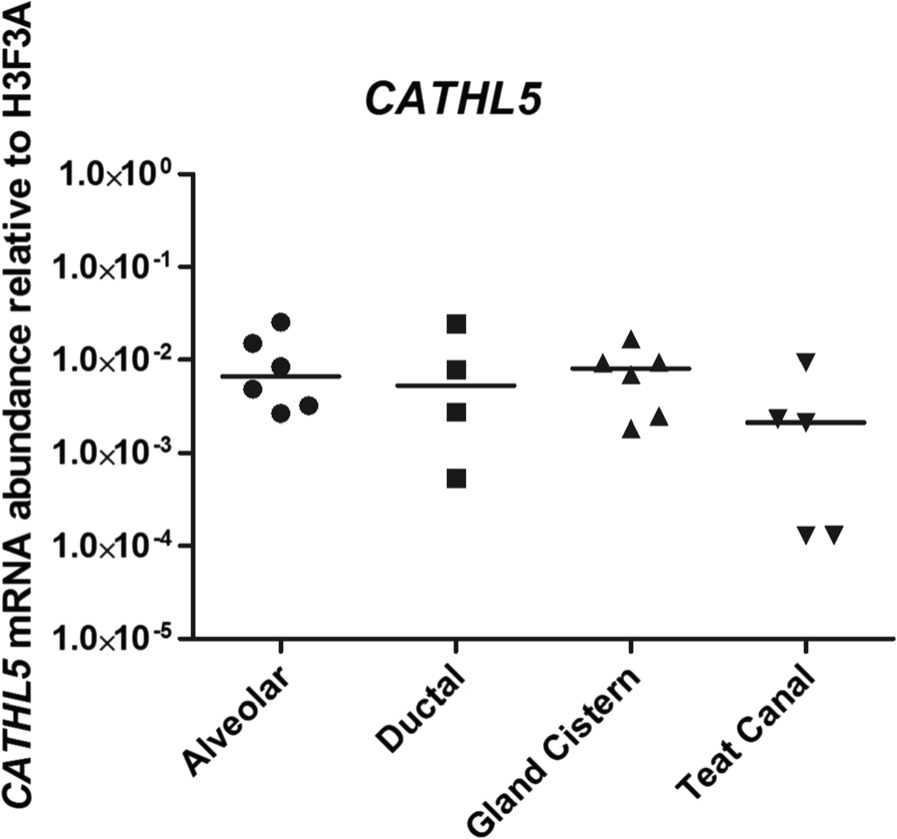
**Expression of *CATHL5 *across several regions in the bovine mammary gland.***CATHL5* mRNA is constitutively expressed in alveolar, ductal, gland cistern and teat canal regions of the bovine mammary gland. Both healthy (and *Staphylococcus aureus-*infected) animals are included; *CATHL5* was not significantly upregulated in the infected group. Samples were collected 48 hours after infection. Results are expressed as a ratio of reference gene expression (H3F3A) in each sample, with mean values indicated by horizontal lines.

We used quantitative real-time PCR (qRT-PCR) to examine the tissue expression patterns of cathelicidins 1-9 in bovine alveolar, ductal, gland cistern and teat canal tissues from the mammary gland of 3 healthy animals and 3 animals which had been experimentally-infected with *Staphylococcus aureus*. Only *CATHL5* mRNA was detected. This was present across all regions in each animal, but did not increase in the infected animals (Figure 6). Once again, none of the samples was positive for *CATHL8* or *CATHL9* using any of the primer sets we designed.

### Cathelicidin gene expression in milk cells from animals with a high somatic cell count

Expression of cathelicidin genes 1-9 was analysed in somatic cells extracted from milk samples taken from 5 cows with a high somatic cell count (Table 2). While six of the seven genes already known to be protein-coding were expressed, expression patterns were highly variable between animals, with no one cathelicidin gene being expressed in all samples. Neither *CATHL8* nor *CATHL9* expression was detected.

**Table 2:** Expression of cathelicidin genes 1-7 in 5 Holstein Friesian cows with high somatic cell count (SCC)

**Sample**	**SCC (cells/ml of milk)**	***CATHL1***	***CATHL2***	***CATHL3***	***CATHL4***	***CATHL5***	***CATHL6***	***CATHL7***
Animal 1	2,249,000	+	-	-	-	+	-	-
Animal 2	1,324,000	+	+	+	-	+	+	+
Animal 3	174,000	+	+	+	-	+	+	+
Animal 4	711,000	-	-	-	+	+	-	-
Animal 5	4,149,000	+	+	+	+	-	-	+

PCR was carried out to detect expression of cathelicidin 1-7 genes in somatic cells isolated from milk samples from cows with a high somatic cell count. - denotes no detectable gene expression, + indicates gene expression was detected.

## Discussion

In this study, we used the most recent assembly of the bovine genome to characterise the cathelicidin gene cluster with a view to reconciling older functional studies with more recent genomic analyses whose results were not fully congruent. It should be noted that these analyses were each carried out on one particular assembly of the genome, of which there have been several, with each being slightly different with respect to the cathelicidin cluster (Figure 2). This search identified seven genes already known to code for functional proteins (cathelicidins 1-7). We also found three additional putative genes whose function is unclear, along with one complete and two incomplete gene duplications clustered on chromosome 22 (Figure 2).

Phylogenetic analysis (Figure 5) indicates *CATHL8*, *CATHL9* and *CATHL3L2* are most similar to *CATHL3*, which gives rise to a linear protein whose proline and arginine-rich motifs are responsible for its antimicrobial function [41]. Gennaro *et al*. found that cathelicidin peptides of this type need at least 16 residues to be functional. *CATHL9* fulfils this criterion, with an antimicrobial domain of 36 residues which contains several proline-arginine repeats [42] (Additional file 2). Despite the sequencing results of this gene indicating a stop codon in exon 1, Scocchi *et al.* were able to detect a transcript in bovine bone marrow RNA via Northern blotting using an oligonucleotide probe based on the antimicrobial domain (exon 4) of this gene [42]. On further examination, this transcript was found to consist of exons 1, 2 and 3 of *CATHL3,* which is adjacent to *CATHL9*, with exons 2, 3 and 4 of *CATHL9.* A peptide based on the antimicrobial domain of *CATHL9* was synthesised and was found to have antimicrobial properties against *E. coli* at low salt concentrations [42]. Therefore *CATHL9* (and perhaps other cathelicidin genes) may extend the bovine cathelicidin repertoire through unusual transcription mechanisms.

Based on the gene expression profiling carried out here, the 7 known protein-coding cathelicidin genes are constitutively expressed in a broad range of tissue types (Table 1), including the mammary gland. Cathelicidin proteins are a major component of the neutrophil secondary granule and increased levels of these proteins are observed in a range of inflammatory conditions [7]. Extensive neutrophil recruitment from the circulation to the lumen of the mammary gland is a hallmark of the early immune response to mammary infection [43]. As an initial step to evaluate the role of the bovine cathelicidins in mammary gland defence, the expression pattern of cathelicidin genes in somatic cells isolated from milk samples was examined. While mRNAs of six of the seven protein-coding cathelicidins were detected, there was no single pattern of expression among the five animals studied (Table 2).

Pryor *et al.*[44] used a pan-cathelicidin antibody targeting the cathelin-like domain along with antibodies targeting cathelicidin proteins 1, 2 and 4 to carry out Western blotting on milk samples from 35 cows with naturally-occurring mastitis involving a range of pathogens. In addition to finding considerable inter-animal variation in the pattern of individual cathelicidin proteins secretion, several samples tested positive with the consensus antibody, yet tested negative with the three specific antibodies. The authors posited that this may be due to the secretion of additional cathelicidin proteins besides those tested. Our results using leukocytes extracted from milk indicate this is probably the case.

The same group have also induced an intramammary *Staphylococcus uberis* infection and measured levels of cathelicidin protein in milk using the antibody specific for targeting the cathelicidin consensus sequence, comparing these results to those from animals with naturally occurring infection [45]. While there was a strong correlation between cathelicidin protein concentration and the progression of infection as measured by somatic cell count in the experimentally-infected animals, the results obtained from animals with naturally-occurring mastitis were less conclusive, as a quarter of animals testing positive for bacterial infection had no detectable cathelicidin protein in their milk.

We hypothesised that our *in vivo* mastitis model may similarly build upon existing data on cathelicidin expression in the mammary gland gathered from naturally infected animals [24]. This model has previously demonstrated that the expression of several innate immune molecules both increases in *Staphylococcus aureus* infected animals and varies across different regions in the gland [46]. However this was not the case for the cathelicidins. *CATHL5* was the only gene whose transcript was detected. While it is constitutively expressed it was not upregulated 48 hours after infection (Figure 6). More extensive analyses of all the cathelicidins across the full spectrum of infection of the mammary gland will be required to determine their effect in this important clinical condition.

It is possible that additional reagents for each HDP may reveal more subtle differences in expression for each cathelicidin. Our demonstration of significant expression of *CATHL5* in mammary tissue as well as milk somatic cells suggests that other cells in addition to immune cells are capable of secreting these potent immunomodulatory molecules.

## Conclusion

In this study, we used the recently released BosTau7 assembly of the bovine genome to reconstruct the full cathelicidin gene repertoire for this species. In addition to cathelicidins 1-7 which are known to code for distinct peptides, three putative cathelicidin genes cluster on chromosome (BTA22); two of these are pseudogenes and one a potentially functional cathelicidin gene. We also identified two *CATHL4* genes adjacent to each other on BTA22. Our *in vivo* data indicate that *CATHL5* is constitutively expressed in the healthy mammary gland, while transcripts of several other protein-coding genes can be detected in leukocytes extracted from the milk of animals with a high somatic cell count.

## Methods

### Characterisation of the bovine cathelicidin gene cluster

Publicly available protein sequences corresponding to the 7 known bovine cathelicidins were retrieved from Uniprot (http://www.uniprot.org/); (Additional file 1A). To carry out Hidden Markov Model [38] searches of the bovine genome for novel cathelicidin genes, the entire bovine genome was translated in all six reading frames using a purpose-written PERL script. To generate accurate HMM models representing the cathelicidin family, the protein sequences of the seven known bovine cathelicidins were aligned using ClustalW, in MEGA5.2 [39]. These sequences were used in the construction of the HMM by the hmmbuild program in HMMER 2.1.1 (http://www.biology.wustl.edu/gcg/hmmerbuild.html). The generated HMM profile was then searched against the translated genome to identify putative cathelicidin-like regions. Chromosomal location and strand orientation of the identified cathelicidins were determined using the BLAST-like Alignment Tool (BLAT) at the University of California, Santa Cruz (UCSC) genome browser (http://genome.ucsc.edu). Genomic DNA corresponding to putative cathelicidins was retrieved using BLAT and used for prediction of intron/ exon boundaries using GenScan (http://genes.mit.edu/GENSCAN.html; [47]).

### Comparison of results with published genomic analyses of the bovine cathelicidin cluster

The three predicted putative cathelicidin sequences located bioinformatically were compared with those published in previous bioinformatic analyses of this gene cluster. Predicted genes described by Elsik *et al.*[35] were downloaded from the Bovine Genome Project database (http://www.bovinegenome.org; [40]). DNA sequences for the putative *CATHL2L*, *CATHL3L1* and *CATHL3L2* genes published by Dawson *et al.*[36] were downloaded from the UCSC genome browser using the co-ordinates for the referenced ENSEMBL sequence tags. These sequences were aligned with our results using ClustalW, in MEGA 5.2 [39], and sequences were defined as being equivalent when there was 100% agreement between two sequences.

### Phylogenetic analysis

The exons from *CATHL1-7*, as well as those for the putative complete cathelicidins, *CATHL8*, *CATHL9* and *CATHL3L2*, were downloaded from the BosTau7 genome assembly on the UCSC genome browser. MEGA5.2 [39] was used to produce a maximum likelihood phylogram. jModelTest2 [48] was used to identify the most appropriate model of sequence evolution for the dataset. A Tamura 3-parameter model with a gamma distribution of substitution rates among sites was chosen. 10,000 bootstrap replicates were run to estimate the reliability of each grouping in the final tree.

### Bovine tissue panel collection

An extensive range of tissues was collected at a local abattoir from recently euthanized healthy cattle and was immediately flash frozen in liquid nitrogen. Each tissue type was collected from an individual animal. These included tissues from the lung, rumen, small intestine, large intestine, testis, uterus, mammary gland, spleen, liver and lymph node. Peripheral blood mononuclear cells were extracted from whole blood of uninfected cattle using a Percoll™ gradient (GE Healthcare UK, Buckinghamshire, UK) and previously described methods [49]. All experimental procedures were carried out under license from the Irish Department of Health and Children in accordance with the European Community Directive 86-609-EC and were approved by the Animal Research Ethics Committee, University College Dublin.

### Bovine mammary gland tissue panel

Our group has previously used an *in vivo* model to study the effects of *S. aureus* infection on the innate immune gene expression in the bovine mammary gland [46, see [50] for detailed description of the model]. Tissues from the unchallenged control animals and from the quarter which was infected 48 hours prior to sample collection in treated animals were used in this study. Bacteriological examination of milk samples taken before the experiment indicated these animals were not infected with any major mastitis-causing pathogen prior to the experiment.

### Extraction of somatic cells from milk samples

Milk samples were collected from a nearby dairy farm. 100 mL volumes of milk were hand-milked from a single quarter in each of 5 Holstein-Friesian cows identified as having a high somatic cell count at the most recent routine count. All samples were collected midway through milking. Samples were processed following the methodology used by Piepers *et al.*[51]. 25 mL volumes of milk were diluted 50:50 in cold phosphate-buffered saline (PBS) (Life Technologies, Dublin, Ireland) before centrifuging at 300 × *g* for 15 minutes at 4°C. Supernatants and fat layers were discarded and cell pellets resuspended in 10 mL of cold PBS. Samples were centrifuged at 4°C for 10 minutes at 200 × *g* before washing the cells once more. Cells were counted using a Z1 Coulter Counter (Beckman Coulter, Co. Clare, Ireland).

### RNA isolation

RNA was extracted from bovine tissue and somatic cells isolated from milk samples using the RNeasy® Mini Kit (Qiagen), and DNAse digested according to the manufacturer’s instructions.

### Quantitative real-time PCR (qRT-PCR)

One μg of total RNA from each sample was reverse transcribed into cDNA using OmniScript™ III first strand synthesis kit with oligo (dT) primers according to the manufacturer’s instructions (Life Technologies). The cDNA was quantified using the ND-1000 NanoDrop® spectrophotometer (Thermo Fisher Scientific, Dublin, Ireland) and then diluted to a 20 ng/μl working concentration. Gene specific primers for quantitative real-time PCR were designed using Primer3 express software. These were designed to traverse introns and then commercially synthesized (Life Technologies) (Additional file 1B). qRT-PCR was performed using the Sybr Green-based fluorescent method and the MX3000P® quantitative PCR system (Agilent Technologies, Cork, Ireland), as follows: 95°C for 10 min, followed by 40 cycles of 95°C for 30 sec, 60°C for 1 min and 72°C for 30 sec, and finally amplicon dissociation at 95°C for 1 min, 55°C for 30 sec and 95°C for 30 sec. Each reaction had a total volume of 25μl: 12.5μl of Brilliant II 2X qPCR low ROX master mix (Agilent Technologies), 10.5μl of the primer-H_2_0 and 2μl of cDNA. Optimal primer concentrations were determined by titrating 100, 250 and 800 nM final concentrations and dissociation curves were examined for the presence of a single product. Amplicons were also assessed on 1.5% native agarose gels to ensure they were the predicted length. Only reactions which yielded a single product with a single band of the correct size were used to determine relative expression levels.

### Data analysis

For differential expression analysis, qRT-PCR data (Cq values) were converted to gene expression fold changes using the 2^-ΔΔCq^ (Cq represents the quantification cycle) method [52], and recorded relative to control samples. GAPDH was used as a reference gene for the multi-organ panel, while H3F3A was previously found to be the most stably expressed from a panel of reference genes tested in the mammary gland samples [46] as determined using Genorm [53].

For baseline expression (expression in control samples) analysis, expression levels of the gene of interest (GOI) was determined as a ratio of the level of expression of the house keeping gene (HK) using the formula (2^-Cq(GOI)^/2^-Cq(HK)^).

Statistical analysis of qRT-PCR results was carried out using the non-parametric Mann–Whitney U test as implemented in version 5.01 of GraphPad Prism (GraphPad Software, San Diego, CA). P values of <0.05 were considered statistically significant.

## Competing interests

The authors declare that they have no competing interests.

## Authors’ contributions

ABR, COF and CJW wrote the manuscript. CJW, ATL and ABR performed the bioinformatic analysis. CJW, ABR and FN prepared the RNA for qRT-PCR. CJW and ABR performed the qRT-PCR experiments and data analysis. PDE provided mammary tissues from the *in vivo* mastitis model. FN, AC and KGM assisted with sample collection and manuscript editing. COF proposed and supervised the study. All authors read and approved the final manuscript.

## Supplementary Material

Additional file 1 **A: Cathelicidin Uniprot Accession numbers. B:** Gene-specific oligonucleotide primers used for qRT-PCR. Click here for file

Additional file 2 **Predicted structure of *CATHL9.*** This figure shows the predicted coding sequence of *CATHL9*, the deduced amino acid sequence and genomic co-ordinates on the Baylor Btau_4.6.1 (BosTau7) assembly. Genomic DNA corresponding to the putative cathelicidin was retrieved using BLAST-like Alignment Tool (BLAT) at the University of California, Santa Cruz genome browser (http://genome.ucsc.edu) and used for prediction of intron/exon boundaries using GenScan (http://genes.mit.edu/GENSCAN.html). The predicted coding sequence is in uppercase letters, noncoding sequences are in lowercase letters. The deduced amino acid sequence of the open reading frame is indicated in single letter code and the stop codon is indicated by an X. Line numbers represent genomic co-ordinates on the BosTau7 assembly. // denotes a break in the sequence. Click here for file

Additional file 3 **Predicted structure of *CATHL3L2.*** This figure shows the predicted coding sequence of *CATHL3L2*, the deduced amino acid sequence and genomic co-ordinates on the Baylor Btau_4.6.1 (BosTau7) assembly. Genomic DNA corresponding to the putative cathelicidin was retrieved using BLAST-like Alignment Tool (BLAT) at the University of California, Santa Cruz genome browser (http://genome.ucsc.edu) and used for prediction of intron/exon boundaries using GenScan (http://genes.mit.edu/GENSCAN.html). The predicted coding sequence is in uppercase letters, noncoding sequence are in lowercase letters. The deduced amino acid sequence of the open reading frame is indicated in single letter code and the stop codon is indicated by an X. Line numbers represent genomic co-ordinates on the BosTau7 assembly. // denotes a break in the sequence. Click here for file

Additional file 4 **DNA sequence of the *CATHL8 *** **promoter region.** This figure shows several potential recognition sites for transcription factors involved in the transcription of immune-related genes in the 5′ flanking of *CATHL8*. Genomic DNA corresponding to the *CATHL8* promoter region (866 base pairs upstream of the translation start codon) was retrieved using BLAST-like Alignment Tool (BLAT) at the University of California, Santa Cruz genome browser (http://genome.ucsc.edu). Predicted transcription binding sites were identified using alibaba2 (http://www.gene-regulation.com/pub/programs.html#alibaba2) and TFSEARCH (http://www.cbrc.jp/research/db/TFSEARCH.html) programs. Several potential recognition sites for transcription factors involved in the transcription of immune-related genes are underlined. Translation start codon ATG is in bold and TATA-box is in italics. Click here for file
