# Supplementary material for: Characterisation and expression profile of the bovine cathelicidin gene repertoire in mammary tissue
Source: BMC Genomics. 2014 Feb 13;15:128. doi: 10.1186/1471-2164-15-128 (PMC3932039; doi:10.1186/1471-2164-15-128)
Supplement: Additional file 1 — A: Cathelicidin Uniprot Accession numbers. B: Gene-specific oligonucleotide primers used for qRT-PCR. [file 1471-2164-15-128-S1.docx]

| **Gene Name** | **Mature host-defence peptide name** | **Uniprot Accession Number** |
| --- | --- | --- |
| *CATHL1* | Bactenecin (Bac)1, cyclic dodecapeptide | P22226 |
| *CATHL2* | Bac5 | P19660 |
| *CATHL3* | Bac7 | P19661 |
| *CATHL4* | Indolicidin | P33046 |
| *CATHL5* | Bovine Myeloid Antimicrobial Peptide (BMAP)-28 | P54229 |
| *CATHL6* | BMAP-27 | P54228 |
| *CATHL7* | BMAP-34 | P56425 |
| *CATHL9* | Bac4 |  |

**Additional file 1b: Gene-specific oligonucleotide primers used for qRT-PCR**

| Gene Symbol | Gene Name | Forward primer (5'-'3) | Reverse primer (5'-'3) | Amplicon size (bp) | Accession Number |
| --- | --- | --- | --- | --- | --- |
| *CATHL1* | Cathelicidin 1 | ATCACCTGTAATAATCACCAGAGCAT | CCCTTAGGACTCTGCTGGCTTA | 150 | NM_174825 |
| *CATHL2* | Cathelicidin 2 | GAGAATGGGCTGGTGAAACAG | GTTATCTGCCTATTGTTCACCGTCTA | 250 | NM_174826 |
| *CATHL3* | Cathelicidin 3 | CAGAAGCTAATCTCTACCGCCT | CTCTGAAGCTCATTACAGTTTAAGT | 238 | NM_174001 |
| *CATHL4* | Cathelicidin 4 | GACCCACCTCCCAAGGATAATG | TTGAAGTCACACTGCTCCGC | 116 | NM_174827 |
| *CATHL5* | Cathelicidin 5 | GGAGAATGGGCTGCTGAAAG | CACAGCACAGGTGATGTCG | 83 | NM_174510 |
| *CATHL6* | Cathelicidin 6 | GGAGGACGATGAGAACCCAAA | AGTAGCGGAATGACTGGAGAAAGT | 270 | NM_174832 |
| *CATHL7* | Cathelicidin 7 | CCCAGAGCAGTGTGACTTCAAG | AGCCCCGCACTCTGAATATTATTA | 120 | NM_174831 |
| *CATHL8* | Cathelicidin 8 | TCAGGGTGAAGGAGACTGTG | ACTCGCCTCATTACAATTCCCT | 150 |  |
| *CATHL8* | Cathelicidin 8 | CGAGCTGAACAGGGAACTTCA | GCCCTGACACTCTGAAGACAG | 71 |  |
| *CATHL9* | Cathelicidin 9 | GAACTTCAGGGTGAAGGAGACC | TCTGAAGCTCATTACAGTTTAGACC | 158 |  |
| *CATHL9* | Cathelicidin 9 | ACTGTAATGAGCTTCAGAGTGTCA | CTTGGCAGTGGTAATGACAATGG | 100 |  |
| *CATHL9* | Cathelicidin 9 | GGAAAATGGGCTGGTGAAACAGTG | CGTTGATGCTGGGGATGAAGTC | 121 |  |
| *GAPDH* | Glyceraldehyde-3-phosphate dehydrogenase | TGAGGACCAGGTTGTCTCCT | GGAGATTCTCAGTGTGGCGG | 273 | NM_001034034.2 |
| *H3F3A* | H3 histone, family 3A | CATGGCTCGTACAAAGCAGA | ACCAGGCCTGTAACGATGAG | 136 | NM_001014389 |
