# Supplementary material for: Characterisation and expression profile of the bovine cathelicidin gene repertoire in mammary tissue
Source: BMC Genomics. 2014 Feb 13;15:128. doi: 10.1186/1471-2164-15-128 (PMC3932039; doi:10.1186/1471-2164-15-128)
Supplement: Additional file 2 — Predicted structure of CATHL9. This figure shows the predicted coding sequence of CATHL9, the deduced amino acid sequence and genomic co-ordinates on the Baylor Btau_4.6.1 (BosTau7) assembly. Genomic DNA corresponding to the putative cathelicidin was retrieved using BLAST-like Alignment Tool (BLAT) at the University of California, Santa Cruz genome browser (http://genome.ucsc.edu) and used for prediction of intron/exon boundaries using GenScan (http://genes.mit.edu/GENSCAN.html). The predicted coding sequence is in uppercase letters, noncoding sequences are in lowercase letters. The deduced amino acid sequence of the open reading frame is indicated in single letter code and the stop codon is indicated by an X. Line numbers represent genomic co-ordinates on the BosTau7 assembly. // denotes a break in the sequence. [file 1471-2164-15-128-S2.pdf]

```
Exon I      52579495  ctggggaccATGGAGACCCAGAGGGCCAGCCTCTCCCTGGGGCGCTGGTCGCTGTGGCTG
                               M··E··T··Q··R··A··S··L··S··L··G··R··W··S··L··W··L·
            52579435  CTGCTGCTGGGACTAGTGCTGCCCTCGGCCAGCGCCCAGGCCCTCAGCTACAGGGAGGCC
                       L··L··L··G··L··V··L··P··S··A··S··A··Q··A··L··S··Y··R··E··A·
            52579375  ATGCTTCGTGCTGTGGATCAGCTCAATGAGCGGTCCTCAGAAGCTCATCTCTACCGCCTC
                      ·M··L··R··A··V··D··Q··L··N··E··R··S··S··E··A··H··L··Y··R··L·
            52579315  CTGGAGCTAGACCCGCCTCCCTAGGACATGAGCTGGGAGGGGACCGGGGACGAGCCTTT
                      ·L··E··L··D··P··P··P··X········································
            52579255  ctcctgcctgctttggccacactgttgccccttcactctggctgtacctcctgtcaggaa
            52579195  ggcacttttccctctaggtgggttcccacctcttccaggaaaccttcccagacctgggtc
            52579135  atctcccagcaccaggcttcctgtcttagcatctctgctgtgggaataggcaccctgcac
                                          //
            52578895  cagcccctgtgaggggaggcgctgccatcagcgctgctgtgagggccgctcctgctctct
            52578835  gtgtgcccgtgaggccgggcacgggctctgtcccctcccctgtgctcccagcaccaagcc
            52578775  cagggccggacacacagtgggctggagaggctgccgtctgggttgggggcagggagacag
Exon II
            52578715  atcagagaaggaaacatgagtgaacctagtttccccactttgactgttgaccagGTGGAG
                                                                            V··E·
            52578655  GACCTGGGAGCTCGAAAGCCTGTGAACTTCAGGGTGAAGGAGACCGTGTGCCCCAGGTCG
                      ·D··L··G··A··R··K··P··V··N··F··R··V··K··E··T··V··C··P··R··S·
            52578595  AACCTGCAGCCCCCAGAGCAGTGTGACTTCAAGGAAAATGGGgtgagcctggggactgag
                      ·N··L··Q··P··P··E··Q··C··D··F··K··E··N··G···················
            52578535  actgagggctgggaataatgcttctcagtgcgagctgaacagggaaatcggaaggtttc
            52578475  cagcatctagagggtgaggtgagcctgggagattatggcccggggggttccagtttgacct
Exon III
            52578415  tgagctacccttccagCTGGTGAAACAGTGTGTGGGGACACTCAGCCTGTACCGGTCTGA
                                      L··V··K··Q··C··V··G··T··L··S··L··Y··R··S··D
            52578355  TGACCCATTCGGTCTAAACTGTAATGAGgtgagtggccctttctgtgttatgcagatgct
                       ··D··P··F··G··L··N··C··N··E································
            52578295  aacaaggtgggttgtggaacatgcttaggaccgatgacccgctgccccatccagggcaga
            52578235  gaaaggccctcctacccgggcccctccctcccccgagccccaggtctccagccctggctc
            52578175  tgcatcccttagagcagtgtttctgtaatgcagtccccaccccggaactgacatgagaca
                                          //
            52577875  aatcatatgcttcaaagataacagccagagggtgaacagggcccaacactcgtggtgtcc
            52577815  cagttagagggctgttcaggtgtgaagtgagggatcttgtcttgacccttgcccagtccc
            52577755  acaacaaatctgttttgtcatggtttacagCTTCAGAGTGTCAGGAGACTTCATCCCCAG
Exon IV                                             L··Q··S··V··R··R··L··H··P··Q
            52577695  CATCAACGTTTCCCAAGGGAAAGGCCATGGCCAAAGCCATTGTCATTACCACTGCCAAGG
                       H··Q··R··F··P··R··E··R··P··W··P··K··P··L··S··L··P··L··P··R
            52577635  CCAGGGCCAAGGCCATGGCCAAAACCCCTGTGATAATCATTGTGAAGGCCaaggtcaagt
                       P··G··P··R··P··W··P··K··P··L··X
            52577575  ttagcgctgtggccaagaccaatgccaagtccacgtccattcctgttgacgtctctcatt
            52577515  ccaaggtgtccttgaaaatggtgaaggattggctgccatcacacccattaaagaatttg
```
