# Supplementary material for: Characterisation and expression profile of the bovine cathelicidin gene repertoire in mammary tissue
Source: BMC Genomics. 2014 Feb 13;15:128. doi: 10.1186/1471-2164-15-128 (PMC3932039; doi:10.1186/1471-2164-15-128)
Supplement: Additional file 3 — Predicted structure of CATHL3L2. This figure shows the predicted coding sequence of CATHL3L2, the deduced amino acid sequence and genomic co-ordinates on the Baylor Btau_4.6.1 (BosTau7) assembly. Genomic DNA corresponding to the putative cathelicidin was retrieved using BLAST-like Alignment Tool (BLAT) at the University of California, Santa Cruz genome browser (http://genome.ucsc.edu) and used for prediction of intron/exon boundaries using GenScan (http://genes.mit.edu/GENSCAN.html). The predicted coding sequence is in uppercase letters, noncoding sequence are in lowercase letters. The deduced amino acid sequence of the open reading frame is indicated in single letter code and the stop codon is indicated by an X. Line numbers represent genomic co-ordinates on the BosTau7 assembly. // denotes a break in the sequence. [file 1471-2164-15-128-S3.pdf]

```
Exon I      52606855  tgagcataaaaggagggtccctcgggctgggaggaggtaggctggggaccATGAAGACTT
                                                                     M··K··T··
            52606795  AGAGGGCCAGCCTCTCCCTGGACGGTGGTCACTGTGGCTACTGCTGCTGGGACTAGCGC
                      X··R··A··S··L··S··L··G··R··W··S··L··W··L··L··L··L··G··L··A··
            52606735  TGCCCTCGGCCAGCGCCCAGGCCCTCAGCTACAGGGAGGCTGTGCTTCGTGCTGTGGATC
                      L··P··S··A··S··A··Q··A··L··S··Y··R··E··A··V··L··R··A··V··D··
            52606675  GCATCAATGATGGGTCCACAGAAGCTCATCTCTACCGCCTCCTGGAGCTAGACCCGCCTC
                      R··I··N··D··G··S··T··E··A··H··L··Y··R··L··L··E··L··D··P··P··
            52606615  CCAAGGATCTCCCTGGAGCCATCTCCCCCCCCCAGCTTTGGCCACACTgtcgccccctt
                      P··K··D··L··P··G··S··H··L··P··P··P··S··F··G··H··T··········
            52606555  cgctcaggctggtcctcctgtcaggagggcacttttccctctgggtgggttcccatctct
            52606495  tccaggaaaccttcccagacctgggggccctcccagcaccaggcttcctgccttagcatct
                                          //

            52606195  tgctgtgagggccgctcctgctttctgtgtgcccgtgaggccgggcacgggctctgtccc
            52606135  ctcccctgtgctcccagcaccaagcccagagcctgacacacagggggctagagaggctgc
            52606075  cgtccgggttgggggcagggagacagatcagagaaggaaacatgagcctgagcccagtct
Exon II
            52606015  ccccactttgatcctcgaccagGTGGAGGACTGGGGAGCTCGAAAGGCTGTGAGCTTCAG
                                            V··E··D··W··G··A··R··K··A··V··S··F··R
            52605955  GGTGAAGGAGACTGTGTGCCCCAGGCCGAGCCTGCAGCCCCCAGAGCAGTGTGACTTCAA
                      ··V··K··E··T··V··C··P··R··P··S··L··Q··P··P··E··Q··C··D··F··K
            52605895  GGAGAATGGGCTGACCTTGGGGGCTGAGACTGAGGGCTGGGATCAATGCTTCTCAGCGCG
                      ··E··N··G··L··T··L··G··A··E··T··E··G··W··D··Q··C··F··S··A··R
            52605835  AGCTGAACAGGGAACTTCAGGGAATgtttccagcccctggcaggtgaggtaagctgagcc
                      ··A··E··Q··G··T··S··G··N···································
Exon III

            52605775  tgggagattatggcccggggtttccagtttgaccttgagctccccttccagCTGGTGAAA
                                                                       L··V··K·
            52605715  CAGTGTTTGGGGACAGTCAGCCTGGACCGGTCCGATGACCAGTTTGACATAAACTGTAAT
                      ·Q··C··L··G··T··V··S··L··D··R··S··D··D··Q··F··D··I··N··C··N·
            52605655  GAGGCGagtggccccttctgtgttgggcatatgctaacagggtgggttgagaaacatcct
                      ·E··A······················································
            52605595  ttggaccaatgacctgctgctccatctagggtagagaaaaggccctcctatctgggccca
            52605535  ccctccccaatccctaggtctccagccctggctctgcatcctttagagaagtggctgtct
                                          //
            52605175  tgatctggggccaaagttcctttggtggctcagtttggggggttgttcatgtggggagaga
            52605115  gtggtcttctcttgacccttgcccagtctcacaagtaatctcttccattgtggttcacag
Exon IV
            52605055  CTTCAGAGTGTCAGGGCTAACtgacctcagcctcccaaacctcggcctcctcacaggcca
                      ·L··Q··S··V··R··A··N··X·····································
            52604995  aggccaaggccacggccatggttcccaccaagattccctggaaaacggtgaaggactggc
            52604935  tatcatacctattaatggcttttggtgaattccgagcctgagggaagcattttaaagata
            52604875  tgatttgttctggatcagacttctggacggtgaaaataaattcttgtgaaaacaacttc
            52604815  ctccaggcttcaatttctattatttccctttttccagcaatggcaccccactccagtact
```
