# Supplementary material for: Characterisation and expression profile of the bovine cathelicidin gene repertoire in mammary tissue
Source: BMC Genomics. 2014 Feb 13;15:128. doi: 10.1186/1471-2164-15-128 (PMC3932039; doi:10.1186/1471-2164-15-128)
Supplement: Additional file 4 — DNA sequence of the CATHL8 promoter region. This figure shows several potential recognition sites for transcription factors involved in the transcription of immune-related genes in the 5′ flanking of CATHL8. Genomic DNA corresponding to the CATHL8 promoter region (866 base pairs upstream of the translation start codon) was retrieved using BLAST-like Alignment Tool (BLAT) at the University of California, Santa Cruz genome browser (http://genome.ucsc.edu). Predicted transcription binding sites were identified using alibaba2 (http://www.gene-regulation.com/pub/programs.html#alibaba2) and TFSEARCH (http://www.cbrc.jp/research/db/TFSEARCH.html) programs. Several potential recognition sites for transcription factors involved in the transcription of immune-related genes are underlined. Translation start codon ATG is in bold and TATA-box is in italics. [file 1471-2164-15-128-S4.pdf]

NF-κB

GCTAGAGGAGAACAAAGCAACCCACTCCAGTATTCTTGCCTGGAGAATCCCATGGATAGT

GAGCCTGGCAGGCCACAGTCCGTAGGGTCTCAAAGAGTCTCAAAGAGTCAGACACGACTG

C/EBPα

AAGCGACTGAGCACACATGCATGCACACATAGCTGATTAACAAATCATGTTATGATAGCT

TCAGGTGAAGAGCAAAGGGACTCAGCCATCTGTATATATGTATCCATTCTCTTCCAACCT

AP-1 GATA

GGAGGCGAACTTCCTAACTGAGAAACAGTGGAGATGAATCCAGGAGAGAGGACAGATGGA

GCCAAGCTCCTGACAGGCTGACGGCTGGTCCCAAAGACAGGATGGTGTAGGGTTGATGGC

TGCCTAGGGACAGGCTGGGTGGGTAAGGCTCAGAAGGGGTTGGTCCTGTCATCCACTCAT

CAAATGTTAAATGAGTGCATACTGTGTGCCAGGCAGTGCCTAGAGCTGGAGGTTGAGTGC

TGAATATACTCCTTTACCCTGAATCCCGAGGAGCCCATGGTGTGGAGGCAGAGGGGATGC

C/EBP

AGACAGTGCCTGGTCCCTCCCTCTCCTGGAGGACACAACCCTCTTGAGCAAGCCTGCCGG

TGACACTCCCCATGGGATCTAGGAGAGGCCAAGAAGTTGTCAGGAGATGGAGAAACCATT

Sp1 NF-κB

TCTTCACCTTGCAAACGGGCCTCCCTGCCCACTGCCCAGAAATCCCATGAGGCAACAGCA

AGGCTTGAGGTAAGACCAGCCCACACCCTGGGAGGAGGGCAGGGATGGGGCGGGTCAGGA

AGACTCCTGGTAGAGCTTTTGCATCAGGGCTCAGACTGGGC*ATAAAA*GAAGGTCCCTTG

GGCTGGGAGGAGGCAGACTCGGGACC **ATG** GAG ACC CAG AGG GCC AGC CTC TCC
